# Electrochemical DABCOylation enables challenging aromatic C–H amination

**DOI:** 10.21203/rs.3.rs-5442169/v1

**Published:** 2025-01-07

**Authors:** Christian Malapit, Griffin Stewart, Eva Maria Alvarez, Chris Rapala, Jonathan Sklar, Julia Kalow

**Affiliations:** Northwestern University; Northwestern University; Northwestern University; Northwestern University; Northwestern University; Northwestern University

## Abstract

The selective amination of aromatic C–H bonds is a powerful strategy to access aryl amines, functionalities found in many pharmaceuticals and agrochemicals. Despite advances in the field, a platform for the direct, selective C–H amination of electronically diverse (hetero)arenes, particularly electron-deficient (hetero)arenes, remains an unaddressed fundamental challenge.^1–10^ In addition, many (hetero)arenes present difficulty in common selective pre-functionalization reactions, such as halogenation^11^, or metal-catalyzed borylation^12^ and silylation^13^. Here, we report a general solution to these limitations that enables selective C–H amination across a comprehensive scope of (hetero)arenes. Key to this strategy’s success is the oxidative generation of highly electrophilic *N*-radical dications from bicyclic tertiary amines (DABCO) that reacts across a wide range of arenes with high selectivity. Notably, this platform constitutes the first anodically generated *N*-radical cations that engage in aromatic C–H amination over well-reported hydrogen atom transfer (HAT) with weak C–H bonds.^14–16^ This C–H amination reaction that allows selective functionalization of both electron-rich and deficient arenes, as well as pyridines, is a rarity in the general area of non-directed aromatic C–H functionalization.^1–4^ This sustainable electrochemical DABCOylation reaction provides access to many complex drug-like aryl- and pyridinylpiperazines with high functionality tolerance, chemoselectivity, and site-selectivity.^17^

## Introduction

**Figure 1:**
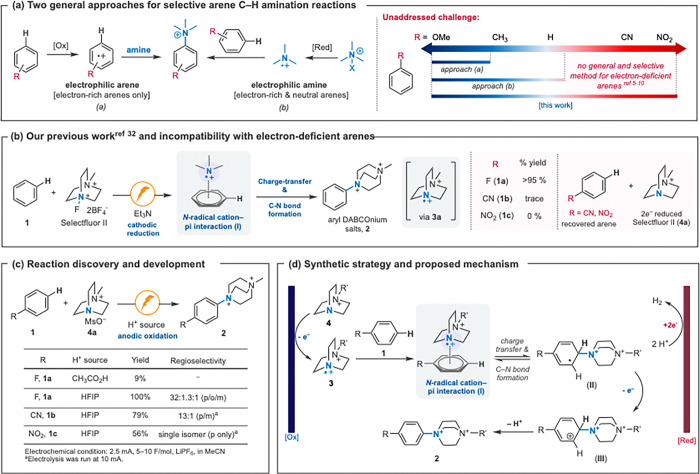
(a) Selective arene C–H amination reactions and challenge associated with electron-deficient arenes. (b) Electron-deficient arenes do not react under reductive C–H DABCOylation reaction.^32^ (c) Discovery and development of an oxidative arene C–H DABCOylation reaction as a general solution to electron-deficient arenes and (d) proposed synthetic and mechanistic strategy.

Aryl nitrogen bonds are ubiquitous across material and medicinal chemistry.^18,19^ About a third of novel small molecule drugs approved by the US Food & Drug Administration in 2023 include an aryl C–N bond.^20^ Aromatic C–H bonds are the ideal substrates for aromatic amination due to their abundance, yet selectivity and substrate scope remain ongoing challenges. Radical redox chemistry has come to fill the void of selective arene C–H amination with two main approaches (Figure 1a).^1–4^ The first such methodology relies on the production of arene radical cations, as pioneered by Yoshida and Nicewicz.^21–23^ In these systems, electron-rich arenes undergo one-electron oxidation, and are then trapped by oxidatively stable sp^2^–*N* nucleophiles to give aryl amines with moderate to high regioselectivity. Second, radical C–H amination based on electrophilic nitrogen radicals was developed, flipping the polarity of the reaction. Most successfully, these radicals have been generated by the reduction of compounds of the general form R_3_N^+^–X (X = F, Cl, RCO_2_, RSO_3_) to form electrophilic *N*-radical (di)cations.^6–10, 24–32^ Selectivity and reactivity for *N*-radical intermediates vary depending on the steric demand and electrophilicity of the radical species and the arene substrate.^1,26^ Despite exploring many *N*-radical scaffolds, these methods have failed to selectively functionalize electron deficient (hetero)arenes (Figure 1a). Past reports of oxidative electrophilic *N*-radical generation have struggled with arene scope due to limited radical electrophilicity.^33–35^

Our group recently published a reductive electrochemical DABCOylation of (hetero)arenes (**1**) utilizing Selectfluor II as the aminating reagent (Figure 1b) to form aryl DABCOnium salts (**2**).^32^ Despite the high electrophilicity of the *N*-Me-DABCOnium radical dication (**3a**), this system struggled to aminate electron-poor systems such as benzonitrile and nitrobenzene. With such arenes, we observed over-reduction of Selectfluor II to *N*-Me-DABCOnium (**4a**) and full recovery of the unreacted arenes (Figure 1b). We hypothesized that the interaction of **3a** with electron-deficient arenes is weak and outcompeted by over-reduction to form **4a** that could no longer participate in amination under reductive conditions. With its high electrophilicity and steric demand, the DABCOnium radical dication (**3a**) should be a promising candidate for selective amination of electron-poor arenes. We hypothesized that if the radical dication **3** was instead sourced from **4a** (rather than Selectfluor) via anodic oxidation, electron-poor reactivity would be unlocked. We therefore envisioned an anodic aromatic C–H amination using a DABCOnium framework to take advantage of the greater reactivity and selectivity of the *N*-radical dication intermediates and the diversification of their products, while also benefitting from the lack of added chemical oxidants with potential substrate incompatibilities.

## Results And Discussion

To discover and develop an arene C–H amination reaction via oxidatively generated *N*-radical cations (Figure 1c), we pursued the mechanistic strategy summarized in Figure 1d. First, *N*-radical cation **3** can be sourced oxidatively from DABCOnium **4** (*E*_p/2_ ~ 1.5 V *vs* Fc/Fc^+^). The bicyclic nature of *N*-alkyl DABCOniums appears to suppress unwanted Shono-type oxidations.^36^ Second, a sacrificial oxidant that undergoes cathodic reduction is needed, which would allow for an operationally convenient undivided electrochemical cell. We hypothesized that proton reduction would meet this requirement. And third, a base that will deprotonate intermediate **III** to form aryl DABCOnium salt **2** is needed. Anodically generated *N*–radical cations from tertiary bicyclic amines are known to undergo hydrogen atom transfer (HAT), as they form strong N–H bonds upon accepting a hydrogen atom.^14–16^ However, DFT calculations have shown that radical addition of **3** into an arene has a lower energy barrier than extracting a benzylic hydrogen atom for electron-neutral arenes under typical amination conditions.^32^

Our initial studies provided promising results using fluorobenzene as the arene and acetic acid as both the sacrificial oxidant and base (as acetate), furnishing the desired product **2a** in 9% yield. Upon further study, we found that HFIP provided much better yields. HFIP is likely superior for two reasons: generated acetate can compete with **4** for anodic oxidation (*E*_p_ = 1.45 V *vs* Fc/Fc^+^ in MeCN)^37^ and HFIP is known to enhance cationic reactivities.^8^ With 1:3 HFIP/MeCN as mixed solvent, LiPF_6_ as supporting electrolyte, and graphite/platinum (anode/cathode) as electrodes, the amination of fluorobenzene to form **2a** was obtained in quantitative yield with high regioselectivity towards the *para*-product (32:1.3:1 *p*/*o*/*m*). Other parameters were also investigated (see SI for details); however, the optimal conditions are shown in Figure 1c.

Free *N*-radical arene C–H amination has previously struggled to selectively aminate electron-poor arenes. Recently, Lei and coworkers published a selective *para*-amination of nitroarenes utilizing proton coupled oxidation of amines. However, this transformation, by design, works only with nitroarenes.^5^ Utilizing our anodic DABCOylation method, a variety of electron-deficient arenes including cyano- (**5b**) and nitro- (**5c**) benzene, triphenyl phosphine oxide (**5d**), trifluorotoluene (**5e**), methyl-2-bromo benzoate (**5f**), and 1,2-dichlorobenzene (**5g**) undergo amination in moderate to quantitative yields with high to singular selectivity. Moreover, the observed selectivity is in agreement with the Fukui index value and thus can be predicted.^32^ Our method has unusually high selectivity for such compounds in free-radical arene C–H functionalization, where functionalization of electron-poor arenes is usually unselective.^6–10,38,39^ To our knowledge, this anodic amination represents the only selective C–H functionalization of benzonitrile with significant diversification potential, as electrophilic halogenation and transition metal-catalyzed borylation/silylation methods are ineffective or unselective on this substrate.^11–13^ Moreover, aryl DABCOnium salts **2** can be isolated as crude solids or can be readily converted to their corresponding aryl piperazines **5**, using an iterative S_N_2/E2 process with potassium cyanide or through use of the aqueous reductant sodium thiosulfate.^26,32^ An X-ray crystal structure of aryl DABCOnium salt **2a** was obtained revealing a comparable aryl C–N bond length (1.49 Å) to related aryl trimethyl ammonium salts (1.52 Å).^40^ This could explain why the reactivity profile of aryl DABCOnium salts is comparable to aryl trimethyl ammonium salts when engaged with transition-metal catalysts.^41^

Pyridines are a privileged class of heterocyclic compounds, and their direct and selective functionalization remains an active goal for reaction development.^42–44^ A variety of halo- and alkyl-pyridine derivatives are amenable to anodic DABCOylation (**5h-l**). Interestingly, the C–H amination takes place selectively at the α-position, even when the substituent does not normally direct for the α-position (**2l**), delivering direct access to 2-pyridinylpiperazine derivatives **5h-l**, a framework commonly found in neurological and antiretroviral drugs, from simple pyridines. Importantly, α-C–H amination of pyridines is a challenging transformation, traditionally accessible only through the Chichibabin reaction.^45^ Hartwig and Fier have modernized pyridine α-C–H amination, leveraging a tandem AgF_2_-mediated fluorination/SNAr type reactivity or Chichibabin type amination through pyridine activation.^42–44^ Meanwhile, modern transition metal-catalyzed borylation^12^ or silylation^13^ of pyridines are selective at the C-3 or C-4 positions. This anodic DABCOylation represents the first electrophilic method for accessing such α-aminated pyridines, with potential for downstream diversification due to the tolerance of halogen substituents.

**Figure 2:**
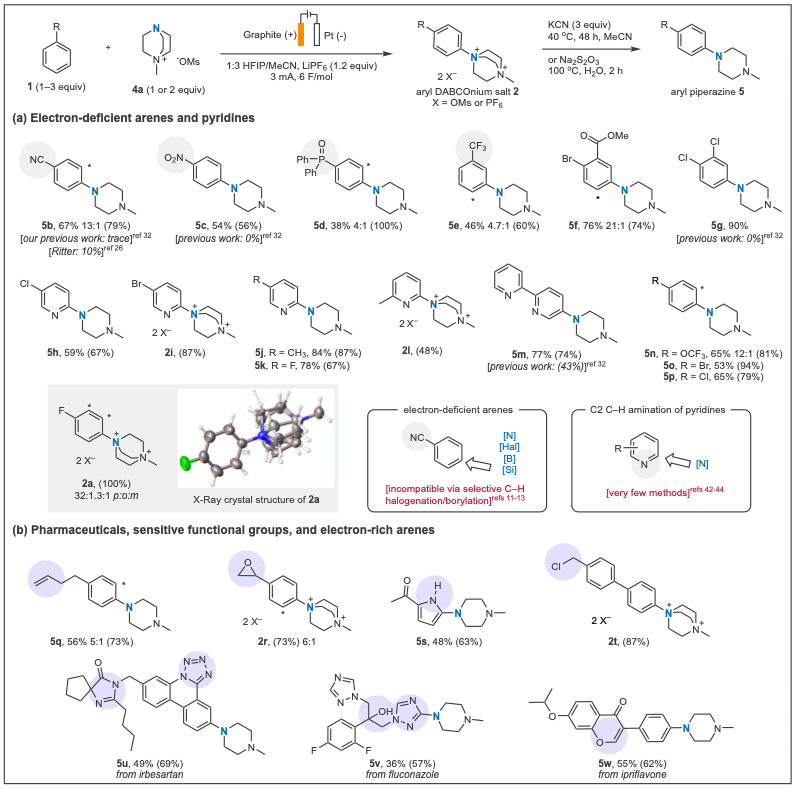
Scope of arene C–H amination reaction. General reaction conditions: **4a** (0.30 mmol, 1.0 equiv.), LiPF_6_ (1.3 equiv.), arene (1.5–3 equiv.), 1:3 HFIP/MeCN (0.075M), graphite anode, platinum cathode, 3 mA constant current electrolysis, 6 F/mol. Yields are isolated yields, values in parentheses are NMR yields of the C–H amination step; for detailed reaction conditions, see SI.

**Figure 3:**
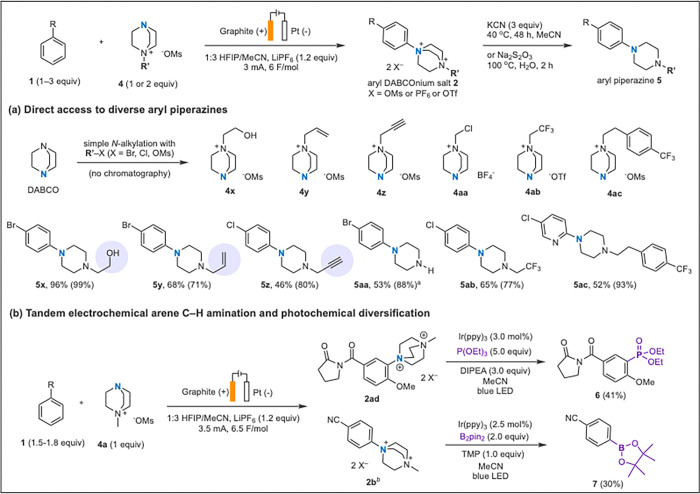
Scope of arene diversification with designer DABCOnium salts (a) and photoredox functionalization of aryl DABCOnium salts (b). Yields are isolated yields, values in parentheses are ^1^H NMR yields, for detailed reaction conditions, see SI. ^a^Obtained from aryl DABCOnium salt resulting from **4aa**; ^b^10 mA, 5 F/mol was used; TMP, 2,2,6,6-tetramethylpiperidine.

Aside from aryl halides many useful and sensitive functionalities were tolerated, such as unprotected alcohols (**5v, 5x**), allylic and benzylic C–H bonds (**5q, 2r, 2t, 5u)**, epoxides (**2r**), benzyl chlorides (**2t**), imines (**5u**), enol ethers (**5w**), and many common *N*-heterocycles, such as pyridines (**5h**–**5m**), pyrroles (**5s**), triazoles (**5v**), and tetrazoles (**5u**). Additionally, to our knowledge, this is the first demonstration of an electrophilic *N*-radical reacting selectively with an arene over an olefin or alkyne, as seen in **5q**, **5y**, and **5z** (Figure 3a).^35,46^ Some free radical arene C–H functionalization methods that are efficacious on electron-poor arenes struggle with electron-rich arenes, due to tendency of the highly electrophilic radicals to participate in SET over π-system addition.^47^ However, this anodic DABCOylation method remains tolerant of electron-rich arenes (Figure 2b) such as anisole (see SI), pyrrole derivatives (**5s**), and biphenyl derivatives (**2t, 5u**).

Another key advantage of this chemistry is its ability to quickly build complexity towards drug-like piperazine compounds. A diverse set of functionalized DABCOnium salts can be used as amine source compared to previous work that is limited to Selectfluor I & II.^26,32^ Using simple S_N_2 alkylation reactions, with no chromatography or crystallization, DABCO was converted to *N*-alkyl DABCOnium salts **4x**–**4ac** containing a diverse set of functional groups.^48^ Subjecting these *N*-alkyl DABCOnium salts to the electrochemical amination reaction, followed by bridge removal, provided easy access to designer aryl piperazines bearing important functional groups such as free alcohols (**5x**), olefins (**5y**), alkynes (**5z**), CF_3_ groups (**5ab**) and other arenes (**5ac**). Tolerance of an alkyne is noteworthy, as they can be used in bio-orthogonal chemistry for *in vivo* applications. In addition, aryl-DABCOnium salts can be utilized in photoredox and transition metal catalyzed diversifications. This includes methylation,^41^ arylation,^32^ phosphorylation (**6**), and borylation (**7**), showing the potential of the aryl DABCOnium as an intermediate for C–C, C–P, and C–B bond formation.

**Figure 4:**
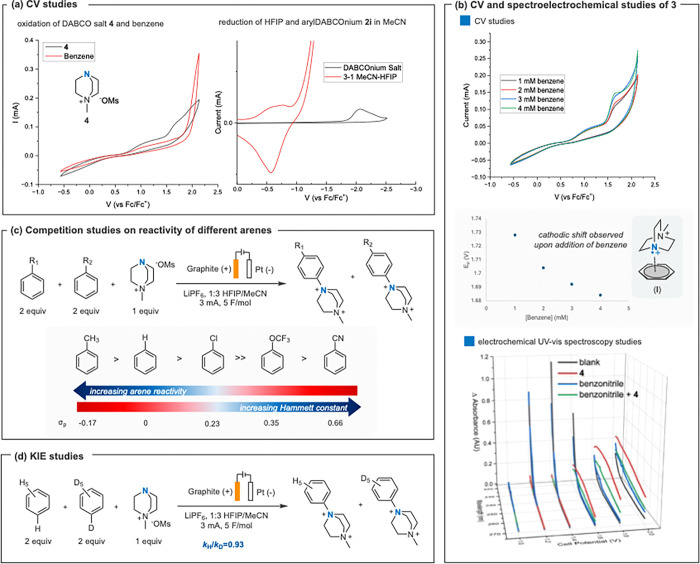
Mechanistic studies. (a) Redox potentials of various reaction components. (b) CV of electrochemically generated dicationic *N*-radical **3** (*via* oxidation of **4**) with benzene and spectroelectrochemical analysis with benzonitrile. The spectroelectrochemical analysis is reported *vs* Ag/AgCl pseudo-reference electrode. (c, d) Competition and KIE studies. See the SI for more details.

To gain insight into the mechanism of this reaction, we performed several experiments including cyclic voltammetry (CV), competition trials, kinetic isotope effect (KIE) experiments, and electrochemical UV–vis spectroscopy (spectroelectrochemistry). CV analysis (Figure 4a) of the reaction components reveal that *N*-alkyl DABCOnium salts **4** undergo accessible oxidation (*E*_*p*/2_ ~ 1.5 V *vs* Fc/Fc^+^) in 3:1 MeCN/HFIP. Moreover, HFIP undergoes proton reduction (*E*_red_ = −1.0 V). while the aryl DABCOnium salt product reduces at more negative potentials (*E*_red_ = −1.8 V). CV studies (Figure 4b) also revealed that upon addition of benzene (Figure 4b) or toluene (see SI) to a solution of **4**, the oxidation profile increases in current and shifts cathodically (44 mV cathodic shift from 1–4 mM benzene). This change is an indication of rapid trapping of the arene, perhaps via an *N*-radical-cation–pi interaction **I**,^32^ leading to faster diffusion away from the working electrode.^49^ In addition, spectroelectrochemical studies provide evidence of the rapid trapping of electrochemically generated dicationic *N*-radical **3** in the presence of benzonitrile, suppressing features associated with oxidation of **4** (Figure 4b). This observation provides evidence for the proposed charge transfer or rapid turnover into radical addition intermediate **II**. The slope of cathodic shift reduces as arene concentration increases, possibly indicating that the arene is saturating almost all generated **3**.

Seeking further clarification of the mechanism, we performed a series of competition experiments. Intermolecular competition between different monosubstituted arenes (Figure 4c)^50^ show that more electron-rich arenes were able to outcompete their electron-poor counterparts. In addition, an intermolecular KIE between benzene and benzene-*d*_*6*_ revealed a *k*_H_/*k*_D_ of 0.93 (Figure 4d). Overall, these experiments rule out C–H bond cleavage as the rate-limiting step and narrows the rate-limiting step to the radical cation–arene interaction or the C–N bond formation. Previously reported *N*-radical C–H amination reactions have proposed C–N bond formation to be rate-limiting by computation.^5^

Overall, we propose a dual mechanistic regime that depends upon the oxidation potential of the arene substrate. The mechanism that enables the selective DABCOylation of electron-deficient arenes and is operational for most shown substrates is summarized is Figure 1c. DABCOnium salt **4** is oxidized on the anode to radical intermediate **3**, which then undergoes trapping by the arene substrate, leading to radical addition intermediate **II**, perhaps through charge transfer complex **I**. Subsequent anodic oxidation to Wheland-type intermediate **III** followed by deprotonation leads to the aryl-DABCOnium salt **2**. Meanwhile, on the cathode, HFIP is reduced to its anion and hydrogen gas via the hydrogen evolution reaction, furnishing the base required for the deprotonation of intermediate **III**. The high site selectivity for most substrates is likely due to the high electrophilicity of the *N*-radical dication and its steric demand.^1,26^ It is also notable that amine **3** is recyclable. When C–N bond formation is challenging, cathodic reduction of **3** can occur, thus regenerating the amine source **4**. That effect, along with the HFIP as co-solvent, likely explains this method’s success with electron-poor arenes where reductive methods have failed.^26,32^

While the above mechanism is probable for electron-poor and -neutral arenes, arenes that oxidize easier than **4** proceed through a direct arene oxidation mechanism. Evidence of this competing mechanism can be seen in the product obtained from irbesartan (Figure 2b, **5u**), which underwent intramolecular amination with its tetrazole moiety (enabled by the oxidation of its biphenyl core) during DABCOylation electrolysis. Other electron-rich arene substrates such as **1s** and anisole (see SI) most likely undergo amination via arene oxidation. This is supported by the spectroelectrochemical data (see SI). In a sample containing anisole and **4**, the oxidation of anisole dominates the spectral features, even at potentials that oxidize **4**. This is unlike similar experiments with more electron-poor benzene and benzonitrile, which show the oxidation of **4** instead. This methodology is particularly advantageous in its mechanistic flexibility, producing a wide range of desired C–H amination products by either the oxidation of **4** or by the direct oxidation of arenes. Since both mechanisms are broadly selective for the most electron-rich site in the molecule, results of arene oxidation in our system are not appreciably different from reductive systems which are always going through *N*-radical mechanisms.^41^ This allows for improved arene scope compared to existing systems.

## Conclusion

In summary, we have developed a general and selective non-directed aromatic C–H amination, effectively addressing a longstanding challenge in late-stage C–H functionalization. In this method, (hetero)arenes that are typically challenging towards C–H amination, classical halogenation, or metal-catalyzed borylation/silylation reactions can effectively undergo C–H DABCOylation with high selectivity. Key to the success of this methodology is the development of an oxidative electrochemical approach to generate highly electrophilic bicyclic *N*-radical dications that rapidly reacts across a wide range of arenes. The electrochemical conditions used bias the generated *N*-radical dications toward aryl reactivity, representing the first oxidatively generated *N*-radical cation that undergoes aryl C–H amination over known reactivity via HAT or olefin addition. The synthetic value of this functionalization strategy is showcased in the rapid construction of many complex drug-like aryl- and pyridinylpiperazines that contain sensitive functionalities such as free alcohols, terminal alkynes, olefins, aryl and alkyl halides, and many common heterocycles. We anticipate that this system can serve as a model for advancing other C–H functionalization reactions that is general and selective for both electron-rich and deficient arenes and amenable to late-stage functionalization.

## Data Availability

All experimental data, copies of spectra, and CIF data are available in the supplementary information.
